# Prolyl Carboxypeptidase Maintains Receptor Tyrosine Kinase Signaling and Is a Potential Therapeutic Target in Triple Negative Breast Cancer

**DOI:** 10.3390/cancers14030739

**Published:** 2022-01-31

**Authors:** Lei Duan, Sarah Calhoun, Ricardo E. Perez, Virgilia Macias, Fatima Mir, Melissa R. Pergande, Paolo Gattuso, Jeffrey A. Borgia, Carl G. Maki

**Affiliations:** 1 Department of Anatomy and Cell Biology, Rush University Medical Center, 600 S. Paulina Ave, AcFac 507, Chicago, IL 60612, USA; lei_duan@rush.edu (L.D.); sarah_j_calhoun@rush.edu (S.C.); ricardo.perez@northwestern.edu (R.E.P.); melissa.pergande@northwestern.edu (M.R.P.); jeffrey_a_borgia@rush.edu (J.A.B.); 2 Department of Pathology, University of Illinois at Chicago, 909 S. Wolcott St, Rm 6128, Chicago, IL 60612, USA; vmacias@uic.edu; 3 Department of Pathology, Rush University Medical Center, Chicago, IL 60612, USA; fatima_mir@rush.edu (F.M.); paolo_gattuso@rush.edu (P.G.)

**Keywords:** prolylcarboxypeptidase, triple negative breast cancer, prognosis, receptor tyrosine kinases, G-protein coupled receptors, targeted therapy

## Abstract

**Simple Summary:**

Triple negative breast cancer (TNBC) is an aggressive cancer type with limited treatment options and poor prognosis. Our research has revealed that a protein called prolylcarboxypeptidase (PRCP) is a potential therapy target for TNBC. We found that high levels of PRCP in tumors coincides with worse prognosis in TNBC patients. Inhibition of PRCP with a small molecule inhibitor blocked TNBC cell and tumor growth and inhibited the activity of several receptor tyrosine kinases (RTKs), proteins that are located on the surface of cells and that are important for cancer development and progression. Our findings suggest that PRCP is a novel prognostic factor for TNBC and that specific inhibitors of PRCP could be developed for TNBC treatment.

**Abstract:**

TNBC is an aggressive cancer sub-type with limited treatment options and poor prognosis. New therapeutic targets are needed to improve outcomes in TNBC patients. PRCP is a lysosomal serine protease that cleaves peptide substrates when the penultimate amino acid is proline. A role for PRCP in TNBC or other cancers, and its potential as a therapy target has not yet been tested. In the current study, we found high tumor expression of PRCP associates with worse outcome and earlier recurrence in TNBC patients. Knockdown of PRCP or treatment with a small molecule PRCP inhibitor blocked proliferation and survival in TNBC cell lines and inhibited growth of TNBC tumors in mice. Mechanistically, we found PRCP maintains signaling from multiple receptor tyrosine kinases (RTKs), potentially by promoting crosstalk between RTKs and G-protein coupled receptors (GPCRs). Lastly, we found that the PRCP inhibitor caused synergistic killing of TNBC cells when combined with the EGFR and ErbB2 inhibitor lapatinib. Our results suggest that PRCP is potential prognostic marker for TNBC patient outcome and a novel therapeutic target for TNBC treatment.

## 1. Introduction

Triple negative (TNBC) breast cancer accounts for 10–20% of all breast cancer cases. TNBC patients have poor prognosis due to the aggressive nature of the tumors, the lack of therapeutic targets, and the high rate of tumor recurrence (metastasis) [1,2]. Identifying novel factors that promote TNBC growth, recurrence, and survival is critical for development of new therapies to combat TNBC [3,4,5].

Multiple receptor tyrosine kinase (RTK) pathways contribute to TNBC growth and progression. These includes the IGF-1R, PDGFR, EGFR, ErbB3 (HER3), FGFR, and c-MET signaling pathways, among others [6,7,8,9,10,11,12]. Ligand binding to RTKs triggers receptor auto-phosphorylation and activation of pathways, such as AKT-mTORC1, MAPK-ERK, and others that promote cancer proliferation, survival, and metastasis (ErbB3 lacks kinase activity and mediates signaling through dimerization with EGFR) The importance of RTK signaling in TNBC was illustrated in a recent study examining PTPN12, a tumor suppressor and phosphatase enzyme that is frequently deleted in TNBC [13]. Loss of PTPN12 causes continuous phosphorylation and activation of multiple RTK signaling pathways and promotes TNBC tumorigenesis. Among the RTKs, IGF-1R, ErbB3, and EGFR have received the most attention in TNBC. High expression or activity of each of these RTKs has been linked in some studies to poor prognosis and/or therapy response [9,14,15,16]. Various small molecule inhibitors of IGF-1R and EGFR have been clinically trialed but have mostly failed to improve TNBC patient outcomes [17,18]. This failure is likely due, at least in part, to continued activation of other RTK signaling pathways.

Prolylcarboxypeptidase (PRCP) is a lysosomal serine protease [19]. PRCP cleaves C-terminal amino acids from peptides when the penultimate amino acid is proline. Peptide cleavage targets of PRCP include α-MSH and angiotensin II [20,21,22], though the full repertoire of cleavage targets is unknown. Very few studies have linked PRCP to cancer. Duan et al. reported that overexpression of PRCP could promote tamoxifen resistance in ER+ breast cancer cells [23]. Our lab reported that in pancreatic cancer cells PRCP maintains levels of IRS1, a protein involved in signaling downstream of IGF-1R and other RTKs [24]. The potential involvement of PRCP in TNBC is unknown.

In the current study, we examined a potential role for PRCP in TNBC cells. High tumor expression of PRCP associates with worse outcome and earlier recurrence in TNBC patients. Knockdown of PRCP or treatment with a small molecule PRCP inhibitor blocked proliferation and survival in TNBC cell lines and inhibited growth of TNBC tumors in mice. Mechanistically, our results suggest that PRCP maintains signaling from multiple RTKs, potentially by promoting crosstalk between RTKs and G-protein coupled receptors (GPCRs). Finally, the PRCP inhibitor caused synergistic killing of TNBC cells when combined with the EGFR inhibitor lapatinib. We propose that PRCP is a viable therapeutic target in TNBC.

## 2. Materials and Methods

### 2.1. Cells and Reagents

MCF7, BT549, MDAMB231, MDAMB468, Hs578T, SUM159PT, BT549, and BT20 breast cancer cell lines were obtained from ATCC (Manassas, VA, USA). MCF7 cells were grown in DMEM medium, the other cell lines were grown in RPMI medium, with 10% fetal bovine serum (FBS), penicillin (100 U/mL), and streptomycin (100 µg/mL). Cells were plated 24 h before treatment with different drugs at the indicated concentrations. Recombinant human EGF, heregulin (HRG), IGF1, angiotensin II (Ang II), and angiotensin 1–7 (Ang 1–7) were obtained from Sigma Chemical Co (St. Louis, MO, USA). Recombinant HGF and FGF were from Abcam. Recombinant PDGF-BB was from Gibco. Trastuzumab, gifitinib, lapatinib, sunitinib, OSI-906, and doxorubicin were obtained from Selleck Chemicals. The PRCP inhibitor (PRCP-7414, referred to as PRCPi in text) was from Calbiochem (catalog number 504044).

### 2.2. Immunoblotting

Whole cell extracts were prepared by scraping cells in lysis buffer (150 mM NaCl, 5 mM EDTA, 0.5% NP40, 50 mM Tris, pH 7.5), resolved by sodium dodecyl sulfate polyacrylamide gel electrophoresis (SDS-PAGE) and transferred to polyvinylidene difluoride (PVDF) membranes (Thermo Fisher Scientific, Waltham, MA, USA). Antibodies to p-EGFR (Y1068), EGFR, p-ErbB2 (Y1221), ErbB2, p-ErbB (Y1289), ErbB3, p-IGF1R (Y1135), IGF1R, p-PDGFRβ (Y751), PDGFRβ, p-Met (Y1234/1235), Met, p-AKT (S473), pan AKT, p-ERK (T202/204), and ERK were from Cell Signaling (Danvers, MA, USA); Phospho-IRS1 (Y612) was from EMD Millipore (Burlington, MA, USA). IRS1 was from Bethyl Laboratories (Montgomery, TX, USA). PRCP was from R&D systems (Minneapolis, MN, USA). β-actin was from Santa Cruz (Dallas, TX, USA). Primary antibodies were detected with goat anti-mouse or goat anti-rabbit secondary antibodies conjugated to horseradish peroxidase (Life Technologies, Carlsbad, CA, USA), using Clarity chemiluminescence (BIO-RAD, Hercules, CA, USA). For some blots, PVDF membranes were cut into halves at the protein markers around 75–100 KD. The upper parts were immunoblotted for high molecular weight proteins, and the lower parts were immunoblotted for low molecular weight proteins.

### 2.3. Flow Cytometry

For cell cycle analysis, cells were harvested and fixed in 25% ethanol overnight. The cells were then stained with propidium iodide (25 µg/mL, Sigma, St. Louis, MO, USA). Flow cytometry analysis was performed on a Gallios™ Flow Cytometer (Beckman Coulter, Brea, CA, USA), analyzed with FlowJo 10 (Treestar Inc., Ashland, OR, USA). For each sample, 10,000 events were collected.

### 2.4. Retroviral and Lentiviral Infection

Human PRCP cDNA in pFB-retroviral vector was co-transfected with packaging vector (pIK) using Fugene (Promega, Madison, WI, USA) into 293FT cells to generate retroviral supernatants. The retroviral supernatants were collected 24 h after transfection and then used to infect subconfluent MCF7 cells as described [23].

The lentiviral pLKO-control shRNA and pLKO-PRCP shRNA were purchased from Openbiosystems (clone ID TRCN50808/50809 for PRCP shRNA1 and PRCP shRNA2, Huntsville, AL, USA) and cotransfected with the lentiviral packaging and envelop vectors psPAX2 and pMD2.G into 293FT cells as described [23]. The lentiviral supernatants were collected 24 h after transfection and used to infect subconfluent BT549, MDA468, MCF7, and BT474_TRLR_ cell lines for 24 h. The cells were then selected with puromycin (2 μM) for 72 h to establish polyclonal lines.

### 2.5. Tissue Microarray Construction and Immunohistochemistry

Primary breast cancer tissues were acquired from archived formalin-fixed, paraffin-embedded (FFPE) pathology tissue blocks in the department of pathology at Rush University Medical Center. For an unbiased analysis, 400 patients consecutively treated from 2000 to 2005 at Rush University Medical Center that had complete follow-up records were selected. For each case, a relevant area of interest was identified on hematoxylin and eosin (H&E) slides and marked, along with the corresponding FFPE tissue blocks, by two pathology residents at the department of pathology. Out of the 400 selected tissue blocks, only 197 cases contained enough intact tumor tissues for construction of TMA. The 197 annotated tissues were submitted to the Pathology Tissue Microarray Core Lab at the University of Illinois at Chicago for tissue microarray (TMA) construction. Two representative cylindrical cores of 1.0 mm in diameter were taken from each donor block and re-embedded into recipient paraffin blocks using a TMA Master arrayer (3D Histech Ltd., Budapest, Hungary), following standard procedures [25]. In total, 394 breast cancer tissue cores and 24 de-identified morphologically benign breast tissue cores arranged as orientation markers were distributed onto four TMA blocks. To increase the adherence of the re-embedded tissue, the recipient blocks were incubated overnight at 37 °C prior to sectioning. Scientific analysis of the cohort of tumors was approved by the Institutional Review Boards at Rush University Medical Center.

The second TMA contains 18 primary tumors of TNBC patients whose tumors recurred after surgical resection. The TMA was obtained through a collaboration with Dr. Elizabeth Wiley in the Department of Pathology at UIC.

The Proteinatlas validated anti-PRCP antibody (HPA017065) was acquired from SigmaAldrich (St. Louis, MO, USA). All the TMA samples were IHC stained with the PRCP antibodies with hematoxylin counterstain using standard procedures at UIC histology core facility. The IHC staining was interpreted by two pathologists, and PRCP positivity was determined for tumors in that more than 60% of the tumor cells have moderate to strong staining patterns.

### 2.6. In Vivo Xenografting and Therapy

NOD.Cg-Prkdc^scid^/J (NOD SCID) and NOD.Cg-Prkdc^scid^Il2rg^tmlWjl^/Sz (NOD-SCID IL2rγ^null^; NSG) mice were obtained from the Jackson Laboratory (Bar Harbor, ME, USA). The mice were maintained under specific pathogen-free conditions in accordance with the ethical guidelines for the care of these mice at the Comparative Research Center of Rush University Medical Center. The mice were 6–8 weeks of age at the time of transplant.

For MCF7 cell transplantation, the mice were subcutaneously inoculated with estrogen pellets (1.5 mg/pellet, 90-day release) obtained from Innovative Research of America (Sarasota, FL, USA) before xenografting. Ten million of disaggregated MCF7 cells or MDA468 cells were resuspended in 100 of a 1:1 *v*/*v* mixture of cold DMEM:Matrigel (BD Biosciences, San Jose, CA, USA) and kept on ice until transplantation. Cells were subcutaneously injected into the left mammary fat pads of NSG (for MCF7) or NOD SCID mice (for MDA468) using 23 G needles. When tumors reached the size of 300 mm^3^, the mice were randomly divided into groups (5 mice/group) for treatment.

For both MCF7 and MDA468 tumors, the mice were treated with a vehicle or PRCPi (20 mg/kg/day or 40 mg/kg/day, 5 days/week). PRCPi was solubilized in Cremophor EL formulation for intraperitoneal injection. Tumor growth and body weight were then monitored with a caliper twice per week. When tumors reached 1 cm^3^ volume, the mice were euthanized. At necropsy, the tumors were harvested for further analysis.

### 2.7. Proliferation Assay with Hoechst Staining of Cellular DNA

Cells were plated in 96-well plates (2 K cells/well) in 8 replicate. The cells were harvested at day 1, day 3, and day 5 in PBS buffer. The cells then underwent three rounds of standard freeze and thaw, followed ny staining with Hoechst 33342 (10 µM in PBS buffer). The fluorescence intensity of Hoechst 33342 was determined using a BioTek (Winooski, VT, USA) Mx microplate reader with an excitation of 361 nm and an emission of 497 nm. Average relative fluorescence intensity (day 1 control conditions are set as 1) of each experimental conditions was presented as graphs with SD indicated.

### 2.8. Statistical Analysis

One-way analysis of variance (ANOVA) and Student’s *t*-test were used to determine the statistical significance of differences among experimental groups. Student’s *t*-test was used to determine the statistical significance between control and experimental groups. To analyze drug synergy, CompuSyn software (downloaded from http://www.combosyn.com/, accessed on 1 July 2021) was used according to the program’s instructions.

## 3. Results

### 3.1. High PRCP Expression Is Associated with Worse Outcome in TNBC

**Figure 1:**
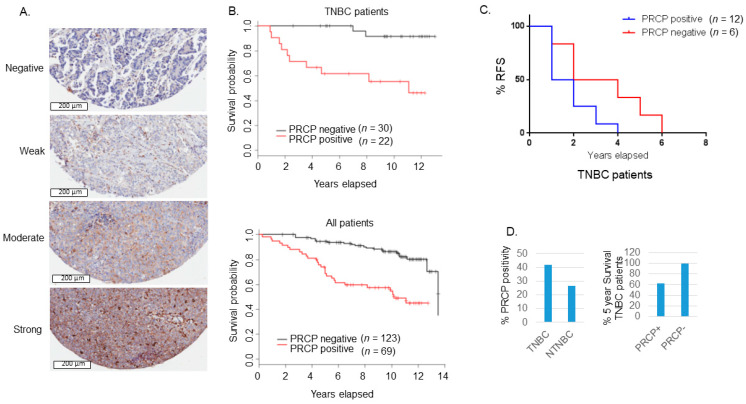
PRCP-positive BC patients have poor outcomes. (**A**) Slides containing cores of breast cancer tissues were deparaffinized and stained with primary PRCP antibody (# HPA017065, Human Protein Atlas). PRCP displayed a granular cytoplasmic pattern. TMA staining was scored based on intensity (negative, weak, moderate, and strong) and percent cell staining. The criteria for PRCP positivity are that more than 60% of the cells have moderate to strong staining. Scale bar: 200 µm. (**B**) Primary breast cancer tissues from 192 patients treated at Rush Medical Center from 2000–2005 were stained for PRCP. Kaplan-Meier curves show overall survival (OS) is significantly lower in PRCP-positive TNBC patients (*p* = 0.0004) (top) and in PRCP-positive breast cancer patients (all patients, bottom) (*p* < 0.0001). (**C**) Primary breast cancer tissues of 18 recurrent TNBC patients treated at UIC from 1994–2004 were stained for PRCP. Kaplan-Meier recurrent free survival (RFS) shows RFS is significantly shorter in PRCP-positive TNBC patients (*p* = 0.041). (**D**) TNBC tumors have higher percentage (41.8%) of PRCP-positivity than non-TNBC (NTNBC, 26.6%). The 5-year survival rate is 61.9% in PRCP-positive TNBC patients, and 100% in PRCP-negative TNBC patients.

To examine a role for PRCP in TNBC, we created a 197 sample tissue microarray (duplicate cores) from breast cancer patients treated at Rush from 2000–2005 and for whom long-term follow-up data is available. This TMA includes 52 TNBCs. The TMA was examined with a validated PRCP antibody and relative PRCP expression blindly scored by pathologists at Rush. The data demonstrated a striking correlation between high PRCP expression and worse patient outcome (decreased overall survival in TNBC patients (*n* = 52) and all breast cancer patients (Figure 1A,B). These findings suggest that PRCP expression can be a prognostic marker in breast cancer, including TNBC. We also examined a second cohort of TNBC patients that developed recurrent tumors and were treated at the University of Illinois-Chicago from 1994–2004. While the cohort was small (*n* = 18), the data nonetheless showed patients whose primary tumors were positive for PRCP staining showed earlier tumor recurrence and reduced recurrence-free survival (Figure 1C). A higher percentage of TNBC tumors scored positive for PRCP expression (42.3%) compared to non-TNBC tumors (NTNBC, 26.6%), and PRCP positivity associated with reduced 5-year survival in TNBC patients (Figure 1D). In total, the results of Figure 1 indicate PRCP positivity is seen in ~40% of TNBC patients and associated with reduced overall and recurrence free survival. We hypothesize these patients could benefit from treatments that target PRCP.

### 3.2. PRCP Promotes TNBC Proliferation and Survival

**Figure 2:**
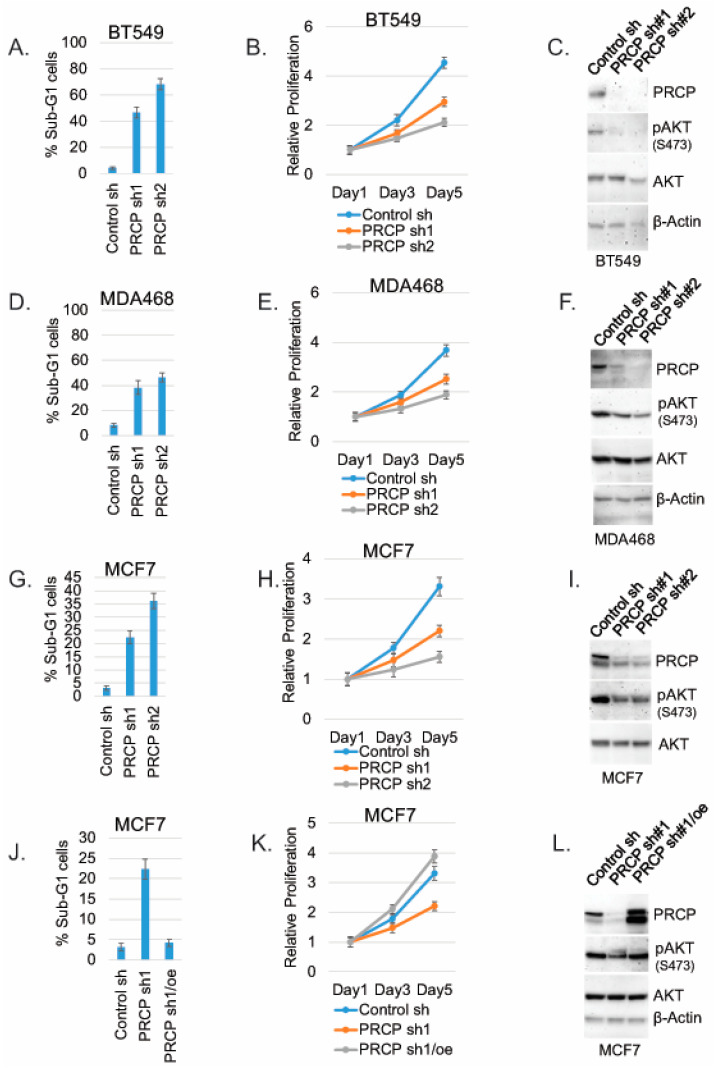
PRCP promotes cell survival, proliferation, and AKT activation. BT549, MDA468, and MCF7 cells were transduced with lentiviral PRCP shRNA (sh1, sh2) or control shRNA. The cells were analyzed with Flow Cytometry for Sub-G1 (apoptosis) at day 5. Average (triplicate) % apoptotic cells is shown (**A**,**D**,**G**). Proliferation was monitored in transduced cells for 5 days by Hoechst staining DNA. Average results from 4 experiments are shown (**B**,**E**,**H**). Lysates were immunoblotted for the indicated proteins (**C**,**F**,**I**). MCF7 cells were transduced with PRCP sh1 alone or together with a WT PRCP cDNA (PRCPre) (**J**–**L**). The cells were analyzed for Sub-G1, proliferation, and immunoblot as described above. Two bands detected in PRCP blots may represent proteolytic cleavage or translation from an alternative translation start site. Original Western Blots and densitometry can be found at Figures S3–S6.

We next investigated the effect of PRCP knockdown on TNBC cell proliferation and survival. To this end, TNBC cell lines MDA468 and BT549 were transduced with lentiviral shRNAs for 24 h to knockdown (KD) PRCP. The cells were selected with puromycin (2 µg/mL) for 72 h, and, at this time, all the mock transduced cells were dead. Similar numbers of control and PRCP shRNA cells were then plated for analysis of proliferation and apoptosis (Sub-G1) and immunoblots. PRCP KD with two different shRNAs (sh1 and sh2) induced apoptosis (evidenced by increased % Sub-G1 cells; Figure 2A,D) and reduced proliferation (Figure 2B,E) in both cell lines. In addition, PRCP KD decreased levels of activated (S473 phosphorylated) AKT in both cell lines (Figure 2C,F), consistent with our previous report that PRCP knockdown reduces activated AKT levels in pancreatic cancer cells [24]. Notably, PRCP KD in MCF7 (ER+) breast cancer cells also induced apoptosis and decreased proliferation and AKT phosphorylation (Figure 2G–I). However, over-expression of PRCP in MCF7 cells blocked shRNA-induced apoptosis, maintained proliferation, and restored AKT phosphorylation (Figure 2J–L), demonstrating the effect of PRCP shRNA on cell apoptosis, proliferation, and AKT is due to depletion of PRCP.

### 3.3. PRCP Inhibitor Can Suppress TNBC Cells In Vitro and Breast Tumor Growth In Vivo

**Figure 3:**
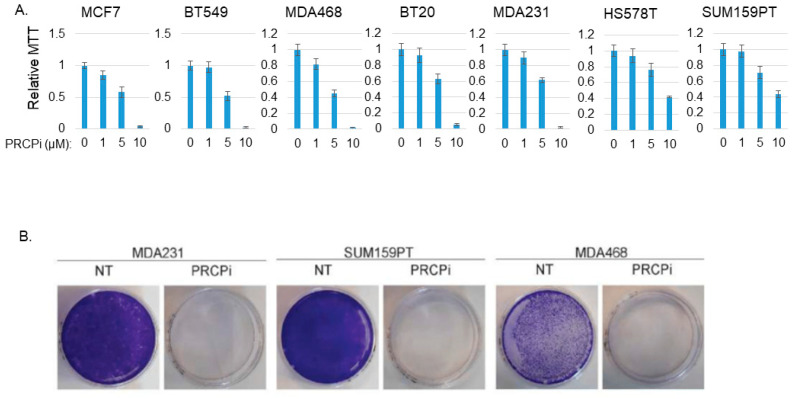
PRCPi decreases TNBC cell viability. (**A**) The indicated MCF7 and TNBC cell lines were treated with vehicle or different doses of PRCPi for three days and viability then analyzed with MTT. Average (8 replicate) relative MTT absorbance is presented, with SD indicated. (**B**) The indicated cells were plated at low density in a P60 dish and, the following day, refed with normal medium or medium containing PRCPi (10 µM). Cell growth was continued for 7 days, at which point the cells on the dish were stained with crystal violet.

**Figure 4:**
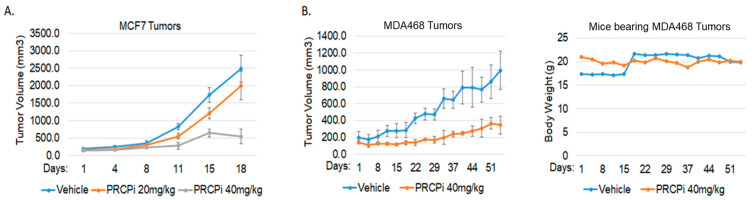
PRCPi suppresses BC tumor growth. (**A**) NSG mice bearing MCF7 tumors were divided to 3 groups and treated daily with a vehicle or PRCPi (20 mg/kg/day or 40 mg/kg/day). Tumor growth (volume) is presented +/− SE. There is a significant difference between the vehicle- and PRCPi- (40 mg/kg) treated mice (*p* < 0.05). (**B**) SCID mice bearing MDA468 tumors were divided to 2 groups and treated daily with a vehicle or PRCPi (40 mg/kg/day). There is a significant difference between the vehicle- and PRCPi-treated mice (*p* < 0.05). Weight of mice during treatment is plotted on the right.

We next asked if a PRCP inhibitor could also block TNBC cell proliferation and survival. For this, we treated BT20, BT549, MDA231, MDA468, Hs578T, and SUM159PT cells, representative of six TNBC subtypes defined by Lehmann et al. [26], with PRCP-7414 (PRCPi), a specific PRCP inhibitor [27]. Cell viability was analyzed by MTT assay after three days treatment and by crystal violet staining after 7 days treatment. As shown in Figure 3A, PRCPi caused a dose-dependent loss of viability as determined by MTT assay in all the TNBC cell lines (Figure 3A), as well as caused loss of viability by crystal violet staining in MDA231, MD1468, and SUM159PT cells (Figure 3B). PRCPi also increased the percentage of sub-G1 cells, which is indicative of apoptosis (Figure S1). PRCP was previously identified in a screen for factors that promote tamoxifen resistance [23]. We tested the ability of the PRCP inhibitor to block growth of tumors formed by MDA468 (TNBC) cells and MCF7 cells, a human breast cancer line that is ER-positive and sensitive to tamoxifen. PRCPi blocked growth of both MDA468 and MCF7 xenograft tumors in mice (Figure 4). The findings support the idea that PRCP contributes to tumor growth and demonstrate that PRCPi has anti-tumor activity in vivo.

### 3.4. PRCP Maintains Signaling from Multiple Receptor Tyrosine Kinases (RTKs)

**Figure 5:**
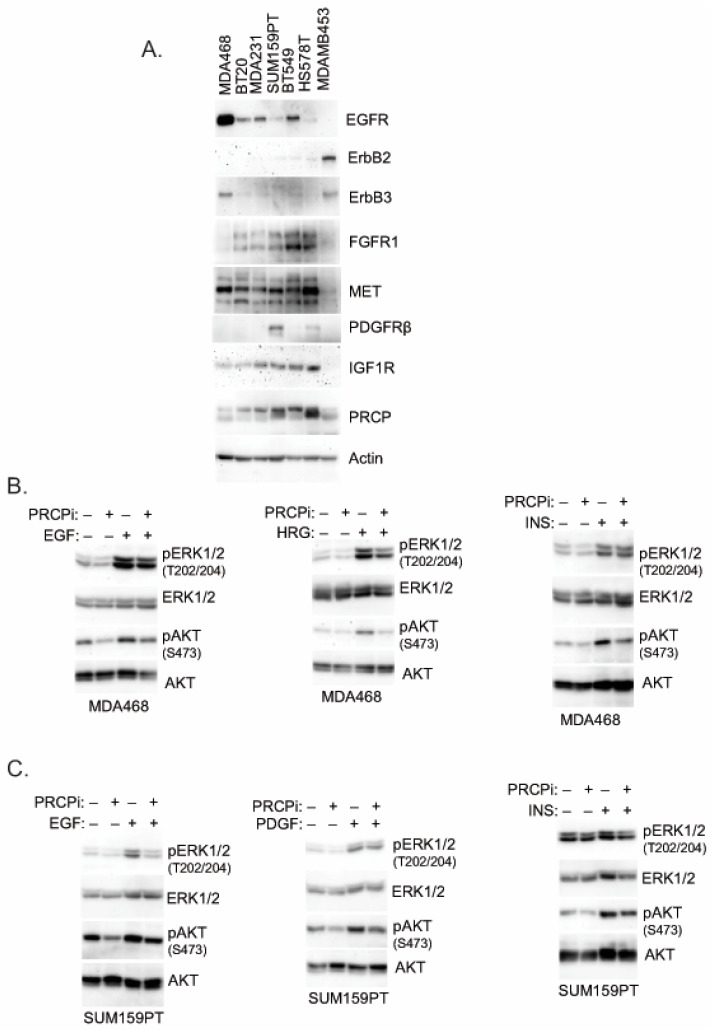
PRCPi decreases RTK ligands-induced activation of AKT and ERK. (**A**) The indicated cell lines were serum-starved overnight, and lysates blotted for the indicated proteins. MDA468 cells (**B**) and SUM159PT cells (**C**) were serum-starved overnight and then treated with EGF (50 ng/mL), Heregulin b1 (HRG, 50 ng/mL), insulin (INS, 50 ng/mL), or PDGF-BB (50 ng/mL) for ten minutes. Lysates were immunoblotted for the indicated proteins. Original Western Blots and densitometry can be found at Figures S7–S9.

**Figure 6:**
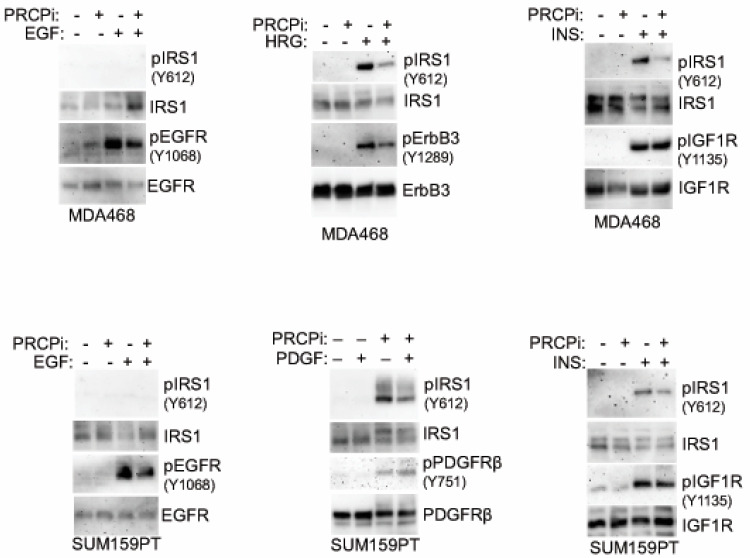
PRCPi decreases tyrosine phosphorylation of RTKs and IRS1. MDA468 cells (top panel) and SUM159PT cells (bottom panel) were serum-starved overnight and then treated with EGF (50 ng/mL), Heregulin b1 (HRG, 50 ng/mL), insulin (INS, 50 ng/mL), or PDGF-BB (50 ng/mL) for 10 min. Lysates were immunoblotted. Original Western Blots and densitometry can be found at Figures S10 and S11.

RTK signaling promotes proliferation, survival, and growth of cancer cells, including TNBC. We previously showed PRCP maintains levels of IRS1, an adaptor protein that functions downstream of IGF-1R and other RTKs [24]. We, therefore, considered PRCP might regulate RTK signaling. The TNBC cell lines express varied levels of different RTKs (Figure 5), reflecting the heterogeneity in TNBCs. MDA468 cells express relatively high EGFR and ErbB3 levels; SUM159PT express high PDGFR but low EGFR and ErbB3. MDA468 and MDAMB453 express relatively low FGFR1 compared to other cell lines, and MDAMB453 express high levels of ErbB2. Notably, PRCP is expressed in all the TNBC cell lines though at different levels (Figure 5A). We note Hs578T and SUM159PT are mesenchymal stem-like [26] and express the highest level of PRCP, while basal-like MDA468 express the lowest level. Moreover, we, and others, have observed that SUM159PT form more rapidly growing tumors than MDA468 [26,28]. Together, the results suggest a correlation between PRCP expression levels and oncogenic potential. To test PRCP’s role in RTK signaling, MDA468 and SUM159PT cells were treated with different RTK ligands, alone or with the PRCP inhibitor. AKT and ERK activation were then used as readouts of RTK signaling. As shown in Figure 5B, PRCPi blocked or reduced AKT activation downstream of EGFR (stimulated with EGF), ErbB3 (stimulated with heregulin (HRG)), IGF1R (stimulated with insulin (INS)), and PDGFR (stimulated with PDGF) in both cell lines, evidenced by reduced levels of activated (S473 phosphorylated) AKT. In contrast, ERK activation (T202/204 phosphorylation) was slightly reduced basally and in response to some ligands by PRCPi, but these effects were less obvious than for AKT activation. The findings suggest that PRCP is required for AKT activation in response to multiple RTKs and may also contribute to ERK activation. We next asked if the PRCP inhibitor effect is due to changes in receptor auto-phosphorylation and/or IRS1 phosphorylation and activation downstream of the receptor. As shown in Figure 6, PRCPi reduced EGFR and ErbB3 phosphorylation in response to the ligand but did not block auto-phosphorylation of IGF-1R and PDGFR. PRCPi also reduced the activating phosphorylation of IRS1 at tyrosine 612 (Y612) in response to heregulin, insulin, and PDGF. EGF did not induce IRS1 phosphorylation at Tyr612. In total, the results support that PRCP maintains signaling from multiple RTKs. PRCPi blocks RTK signaling by reducing RTK auto-phosphorylation (or trans- phosphorylation in the case of ErbB3) and reducing RTK-mediated phosphorylation and activation of IRS1.

### 3.5. PRCP May Promote RTK Signaling via Protein Kinase A (PKA) and Calmodulin-Dependent Protein Kinase II (CaMKII)

**Figure 7:**
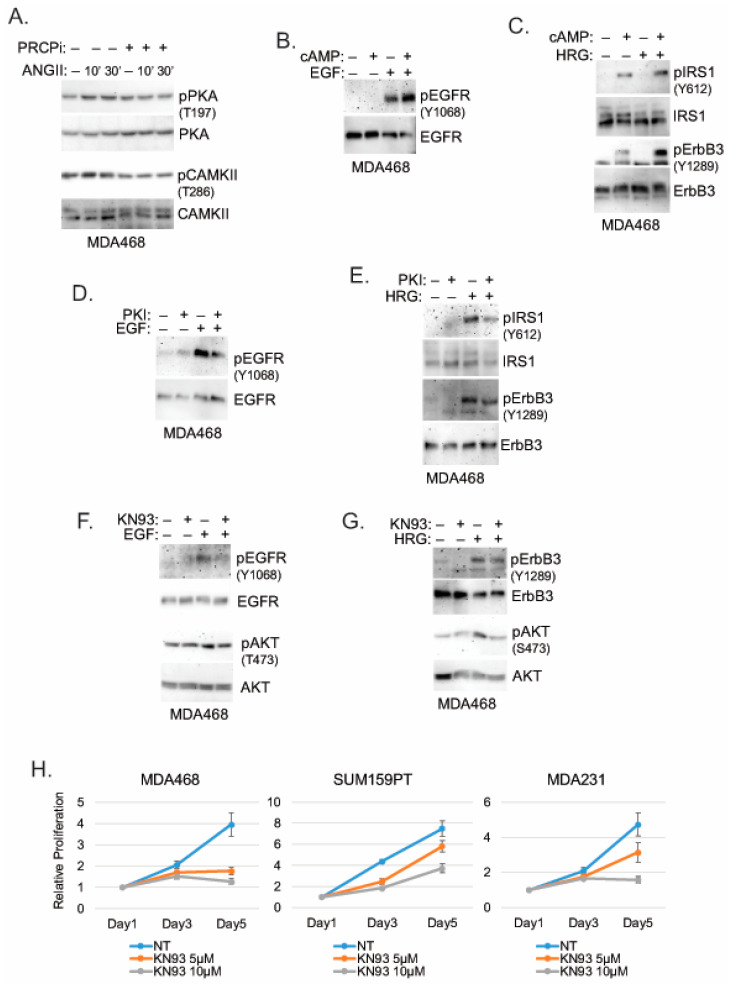
PRCPi suppresses PKA and CAMKII activation to decrease activation of EGFR/ErbB3 and IRS1. (**A**) MDA468 cells were serum-starved and pretreated with a vehicle or PRCPi (10 µM) and then treated with Ang II (100 nM) for the indicated times. Serum-starved MDA468 cells were treated with cAMP and/or EGF (**B**), cAMP and/or HRG (**C**), PKI and/or EGF (**D**), PKI and/or HRG (**E**), KN93 and/or EGF (**F**), or KN93 and/or HRG (**G)** for 10 min. Lysates were immunoblotted for the indicated proteins. (**H**) The indicated cell lines were cultured in the presence of the vehicle or KN93 (5 µM and 10 µM) and were harvested at the indicated times. Relative cell proliferation was analyzed with Hoechst staining of cellular DNA. Average (8 replicate) relative fluorescence intensity of Hoechst is presented with SD indicated. There is significant differences (*p* = 0.000) between the vehicle and 10 µM KN93 conditions in all cell lines. There is a significant difference (*p* = 0.000) between the vehicle and 5 µM KN93 in MDA468 cells. There are no significant differences (*p* > 0.1) between the vehicle and 5 µM KN93 conditions in SUM159PT and MDA231 cells. Original Western Blots and densitometry can be found at Figures S12–S14.

We sought the potential mechanism by which PRCP may regulate RTK phosphorylation and activity. PRCP cleaves substrate peptides, such as Angiotensin II (Ang II) and α-MSH, which activate signaling through their cognate G-protein coupled receptors (GPCRs). Protein kinase A (PKA) and Calmodulin-Dependent Protein Kinase II (CaMKII) are serine/threonine kinases activated downstream of GPCRs [29,30]. Notably, both PKA and CaMKII have been implicated in the activation of RTKs and stabilization/activation of IRS1 [31,32,33,34,35]. We, therefore, speculated PRCP may promote PKA and CaMKII activation to regulate RTKs. Overexpression of PRCP in MCF7 cells increased Ang II cleavage and increased activating phosphorylations in PKA (T197) and CaMKII (T268) basally and in response to Ang II treatment (Figure S2), supporting the idea that PRCP promotes or maintains PKA and CaMKII activity. Ang II treatment in MDA468 cells increased activating phosphorylations in PKA (T197) and CaMKII (T268), which was blocked by co-treatment with PRCPi (Figure 7A). This supports the idea that PRCP maintains or promotes PKA and CaMKII activity in TNBC cells. Lastly, we asked if PKA and CaMKII are required for RTK signaling. MDA468 cells were treated with RTK ligand (HRG, EGF), alone or in combination with PKA activator (cAMP) or PKA inhibitor (PKI) or CaMKII inhibitor (KN93). As shown in Figure 7B,C, EGF- and HRG-induced phosphorylation/activation of EGFR, ErbB3, and IRS1 is increased by cAMP and blocked/reduced by both PKA inhibitor (Figure 7D,E) and CaMKII inhibitor (Figure 7F,G). EGF- and HRG-induced phosphorylation/activation of AKT is also reduced by KN93 (Figure 7F,G). Finally, KN93 reduced proliferation in TNBC cells (Figure 7H), which further supports the importance of CAMKII in mediating PRCP oncogenic activity. In total, the results indicate PKA and CaMKII are required for or promote RTK activation and signaling in TNBC cells. PRCP appears to promote RTK signaling by maintaining PKA and CaMKII activation and crosstalk between GPCRs and RTKs.

### 3.6. PRCP Inhibitor Synergizes with Lapatinib to Kill TNBC Tumor Cells

**Figure 8:**
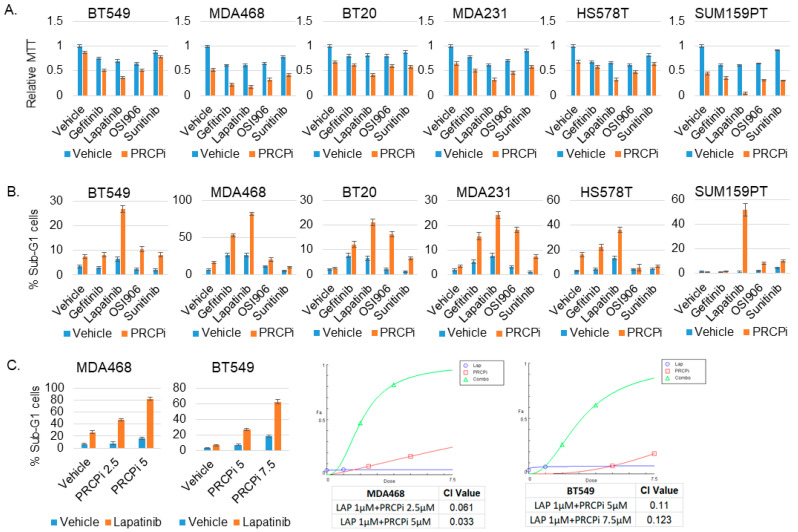
PRCPi and lapatinib synergistically kills TNBC cells. The indicated cell lines were treated with a vehicle, PRCPi (5 μM for 5 cell lines, 10 μM for SUM159PT), Gefitinib (0.5 μM), Lapatinib (2 μM), OSI906 (2 μM), and Sunitinib (0.2 μM), alone or combination of PRCPi with Gefitinib, Lapatinib, OSI906, or Sunitinib, for three days. Cells were analyzed for MTT absorbance (**A**) or % Sub-G1 cells (**B**). Average (8 replicate) relative MTT absorbance and Average (triplicate) % Sub-G1 cells are presented with SD indicated. (**C**) To determine drug synergy, cells were treated with two different doses of PRCPi (2.5 μM and 5 μM for MDA468. 5 μM and 7.5 μM for BT549), alone or in combination with lapatinib (2 μM). % sub-G1 cells was presented (left panels). Combination index (CI) values were calculated with CompuSyn and presented (right panels). Note that CI value < 1 indicates drug synergy.

Because PRCPi inhibited signaling from all RTKs tested, we considered it may synergize with agents that inhibit one or a few individual RTKs. To test this, we measured viability (MTT absorbance) and apoptosis (%Sub-G1 cells) in TNBC cells treated with PRCPi and different RTK inhibitors. To keep our studies clinically relevant, we included RTK inhibitors (lapatinib, gefitinib, sunitinib) that are FDA approved for breast or other cancers. In addition, we only used RTK inhibitor doses at or near that which can be achieved in clinical patients (c_max_). For example, the c_max_ for lapatinib is ~4 μM, and we used 2 μM; the c_max_ for gefitinib is ~0.4 μM, and we used slightly higher 0.5 μM; the c_max_ for sunitinib is ~0.2 μM, and we used 0.2 μM [36]. OSI-906 (linsitinib) was also included though it is not FDA approved. Lapatinib inhibits EGFR and ErbB2, Sunitinib inhibits VEGFR, PDGFR, FLIT3, RET, CSF1R, Gefitinib inhibits EGFR, and OSI-906 inhibits IGF-1R and Insulin receptor. Two things became apparent. First, by MTT assay (Figure 8A), lapatinib in combination with PRCPi caused the most pronounced reduction in viability in 5/6 cell lines (in MDA468 both lapatinib and gefitinib in combination with PRCPi caused a pronounced reduction). Second, by apoptosis assay (Figure 8B,C), lapatinib in combination with PRCPi caused a pronounced, synergistic increase in % sub-G1 cells in all the cell lines. MDA468 cells were most sensitive to combination lapatinib plus PRCPi, showing apoptosis in ~80% of cells, followed by SUM159PT cells which showed apoptosis in ~50% of cells (Figure 8). The results suggest that combined inhibition of PRCP and EGFR family members promotes death in TNBC cells.

## 4. Discussion

PRCP is a lysosomal serine protease that cleaves peptide substrates when the penultimate amino acid is proline. PRCP has been linked to blood pressure and appetite control through cleavage of peptide substrates, such as angiotensin II and αMSH. A role for PRCP in TNBC or other cancers has, to date, not been widely appreciated. In the current study we found high tumor expression of PRCP is associated with worse outcome and earlier recurrence in TNBC patients. shRNA-mediated knockdown of PRCP or treatment with a small-molecule inhibitor of PRCP reduced TNBC cell survival and proliferation and blocked TNBC tumor growth in mice. RTK signaling promotes proliferation, survival, and growth, and we found that the PRCP inhibitor blocks signaling from multiple RTKs to AKT. This offers a potential mechanism for how PRCP maintains TNBC proliferation and survival. Finally, we found the PRCP inhibitor caused a synergistic killing of TNBC cells when combined with the current FDA-approved drug lapatinib, which targets EGFR and ErbB2. There are currently no targeted therapies for TNBC treatment. Our findings suggest that PRCP expression is a potential prognostic marker for outcome in TNBC patients, as well as support the potential use of PRCP inhibitors, alone or in combination with lapatinib or other agents, for treatment of TNBC tumors.

We found PRCP inhibition blocked activation/phosphorylation of ErbB3 and EGFR in response to their respective ligands EGF and HRG. In contrast, the PRCP inhibitor did not block activation/phosphorylation of IGF-1R or PDGFR in response to their ligands insulin or PDGF but did block the activation/phosphorylation of IRS1 downstream of insulin or PDGF. These results suggest that PRCP maintains RTK signaling by either promoting activation of the receptor itself or activation of IRS1 downstream of the receptor. Still, the mechanism by which PRCP promotes phosphorylation and activation of RTKs and/or IRS1 is unclear. The peptide substrates of PRCP (e.g., angiotensin II, α-MSH) are GPCR agonists that promote signaling downstream of their cognate receptors. PKA and CaMKII are activated downstream of GPCRs and have been implicated in the activation of EGFR and stabilization and activation of IRS1. Therefore, we speculated PRCP may promote RTK signaling by maintaining PKA and/or CaMKII activity. Consistent with this, overexpression of PRCP caused greater activation of PKA and CaMKII in response to Angiotensin II (Ang II), while the PRCP inhibitor blocked PKA and CaMKII activation in response to Ang II. Moreover, PKA and CaMKII inhibitors blocked or reduced EGFR, ErbB3, IRS1, and AKT activation in TNBC cells in response to EGF and HRG treatment. In total, the results suggest that PRCP may promote RTK signaling by maintaining PKA and CaMKII activity and promoting crosstalk between GPCRs and RTKs. It is noteworthy that PRCP was identified as tamoxifen resistance factor in ER+ MCF7 cells [23]. Findings in the current study that high tumor expression of PRCP correlates with worse outcomes in TNBC patients suggest its ability to promote RTK signaling may lead to more aggressive tumors, as well as further indicate the prognostic potential of PRCP is not limited to ER+ cancers.

PRCPi caused synergistic killing of TNBC cells when combined with the dual EGFR/HER2 inhibitor lapatinib. Notably, ErbB3 (HER3) lacks intrinsic kinase activity and mediates signaling through dimerization with EGFR or HER2, and ErbB3 signaling, therefore, can also be inhibited by lapatinib. We hypothesize the ability of the PRCP inhibitor to cause synergistic killing when combined with lapatinib results from blocked signaling from multiple RTKs in the combined inhibitor-treated cells. Neratinib is the latest generation HER-family inhibitor that was recently FDA approved, and it will be interesting to test if the PRCP inhibitor also causes synergistic TNBC cell killing when combined with neratinib. Notably, MDA468 cells express high levels of EGFR and ErbB3 and were most sensitive to the PRCP inhibitor, alone or in combination with lapatinib (SUM159PT cells were also sensitive to PRCPi plus lapatinib when PRCPi was used at the 10 micromolar dose; 5 micromolar PRCPi did not cause synergistic killing in SUM159PT cells when combined with lapatinib but did cause synergistic killing of MDA468 cells.). This raises the possibility that TNBC cells with high EGFR and/or ErbB3 expression may be especially sensitive to combined PRCP inhibitor plus lapatinib or neratinib treatment.

The PRCP inhibitor blocked or reduced growth not only of TNBC cell (MDA468) tumors in mice but also the growth of ER+ MCF7 cell tumors. These findings indicate PRCPi is bioavailable with anti-tumor activity, as well as further indicate that the potential therapeutic use of PRCP inhibitors extends beyond TNBC. It will be important in the future to explore PRCP expression in other cancer types and to identify PRCPi drug combinations that can most effectively inhibit tumor growth.

## 5. Conclusions

PRCP is a potential prognostic marker for TNBC patient outcome and a novel therapeutic target for TNBC treatment.

## Data Availability

All images and raw data available on request. All other data are available from the corresponding authors.

## Supplementary Materials

The following supporting information can be downloaded at: https://www.mdpi.com/article/10.3390/cancers14030739/s1, Figure S1: PRCPi induces apoptosis in TNBC cell lines. The indicated cell lines were treated with vehicle or PRCPi (5 μM or 10 μM) for 72 h. The cells were analyzed with FACS for cell cycle. Average % sub-G1 cells were presented with SD indicated; Figure S2: PRCP cleaves Ang II and increased Ang II-mediated phosphorylation of PKA and CAMKII. A. MCF7 and B6-9 cells were serum starved and treated with Ang II (100 nM) for 5 h. Peptides were then precipitated from media and quantitatively analyzed for processed peptide products. Note that Ang (1–7) is increased in B6-9 cells compared with MCF7 cells. B. MCF7 and B6-9 cells were serum starved and treated with Ang II (100 nM) for 10 min. Lysates were immunoblotted for the indicated proteins; Figure S3:Original immunoblots for Figure 2C; Figure S4: Original immunoblots for Figure 2F; Figure S5: Original immunoblots for Figure 2I; Figure S6: Original immunoblots for Figure 2L; Figure S7: Original immunoblots for Figure 5A; Figure S8: Original immunoblots for Figure 5B. Note that PVDF membranes were cut into; Figure S9: Original immunoblots for Figure 5C. Note that PVDF membranes were cut into; Figure S10: Original immunoblots for Figure 6A. Note that PVDF membranes were cut into; Figure S11: Original immunoblots for Figure 6B. Note that PVDF membranes were cut into; Figure S12: Original immunoblots for Figure 7A; Figure S13: Original immunoblots for Figure 7B–E. Note that PVDF membranes; Figure S14. Original immunoblots for Figure 7F,G. Note that PVDF membranes were cut into halves and the upper parts were used for EGFR and ErbB3 blots and lower parts were used for AKT blots.
