# Supplementary material for: Prolyl Carboxypeptidase Maintains Receptor Tyrosine Kinase Signaling and Is a Potential Therapeutic Target in Triple Negative Breast Cancer
Source: Cancers (Basel). 2022 Jan 31;14(3):739. doi: 10.3390/cancers14030739 (PMC8833515; doi:10.3390/cancers14030739)

Original Blots

Figure S3. Original immunoblots for Figure 2C.

Figure S4. Original immunoblots for Figure 2F.

Figure S5. Original immunoblots for Figure 2I.

Figure S6. Original immunoblots for Figure 2L.

Figure S7. Original immunoblots for Figure 5A.

Figure S8. Original immunoblots for Figure 5B. Note that PVDF membranes were cut into halves and the lower parts were used for the indicated blots.

Figure S9. Original immunoblots for Figure 5C. Note that PVDF membranes were cut into halves and the lower parts were used for the indicated blots.

Figure S10. Original immunoblots for Figure 6A. Note that PVDF membranes were cut into halves and the upper parts were used for the indicated blots.

Figure S11. Original immunoblots for Figure 6B. Note that PVDF membranes were cut into halves and the upper parts were used for the indicated blots.

Figure S12. Original immunoblots for Figure 7A.

Figure S13. Original immunoblots for Figure 7B, 7C, 7D, 7E. Note that PVDF membranes were cut into halves and the upper parts were used for the indicated blots.

Figure S14. Original immunoblots for Figure 7F, 7G. Note that PVDF membranes were cut into halves and the upper parts were used for EGFR and ErbB3 blots and lower parts were used for AKT blots.

Figure S3

Figure 2C original blots

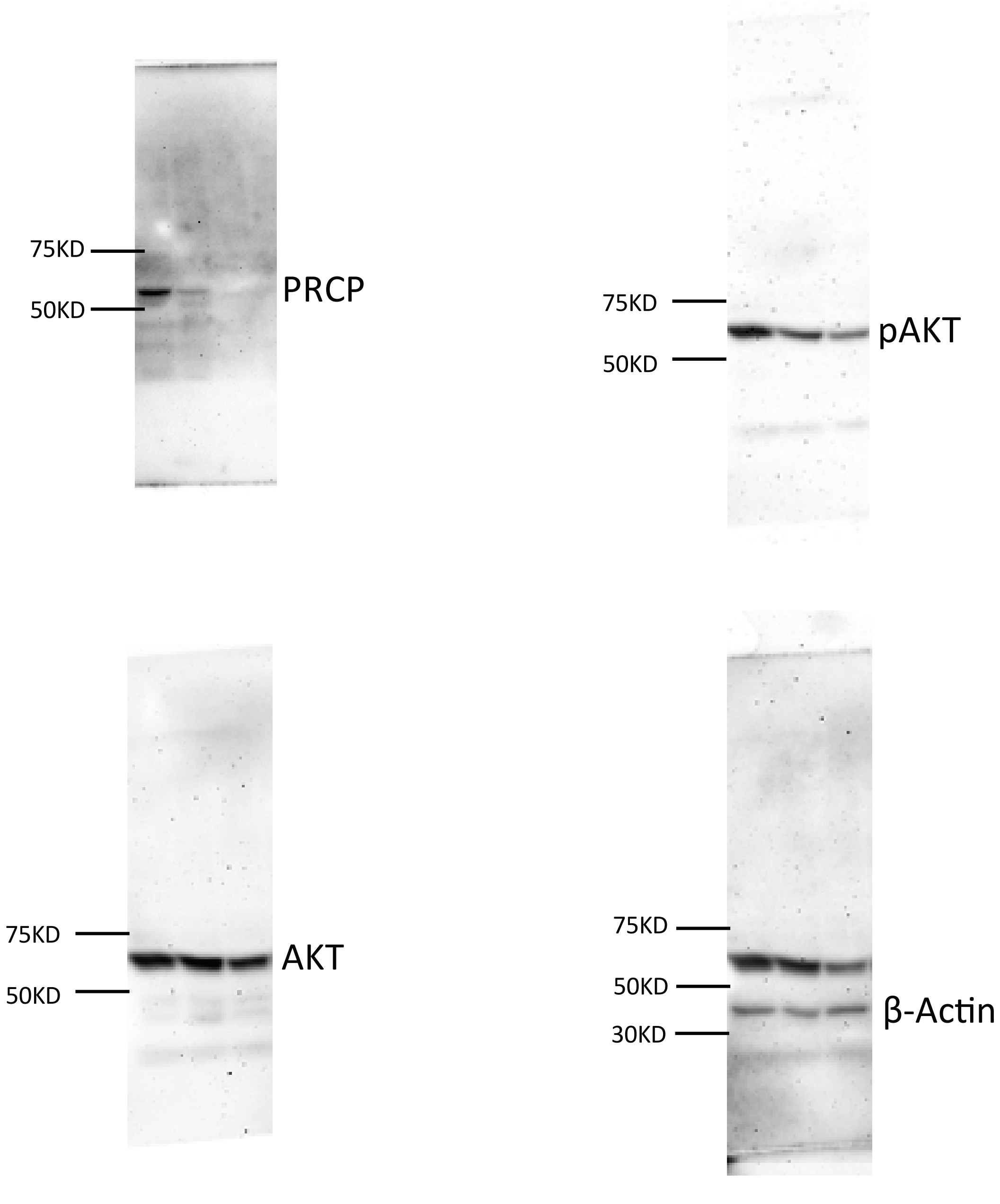

Figure S4

Figure 2F original blots

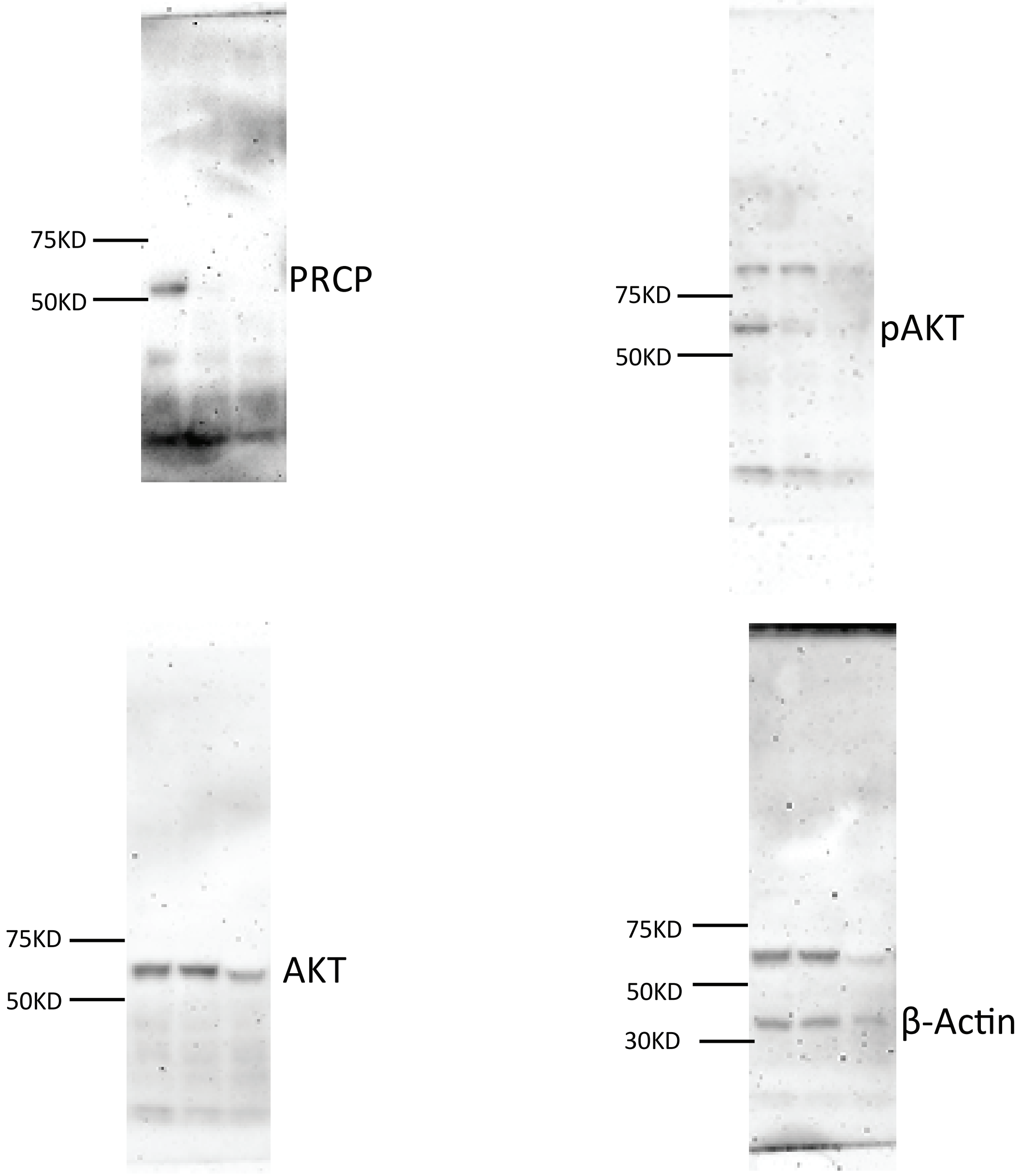

Figure S5

Figure 2I original blots

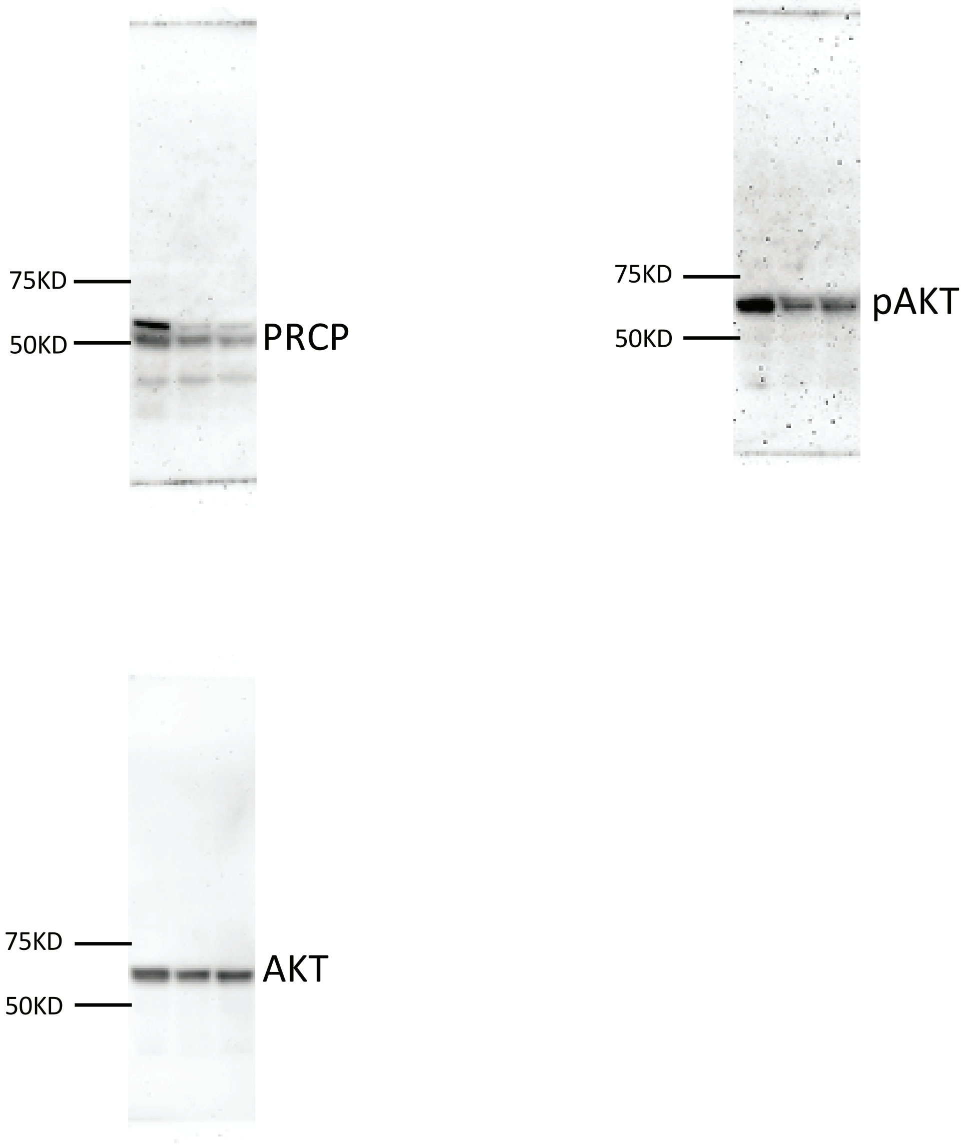

Figure S6

Figure 2L original blots

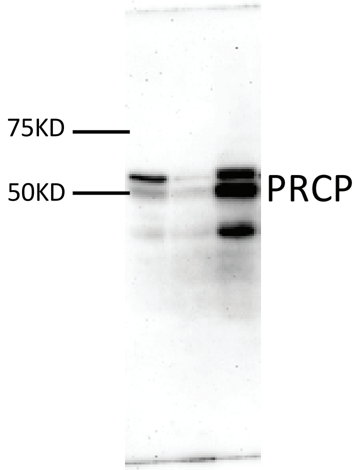

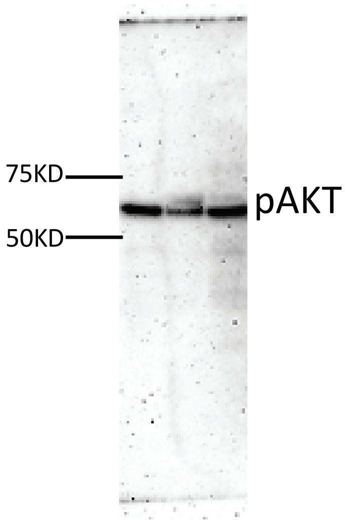

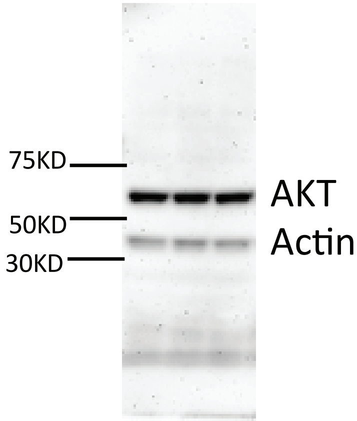

Figure S7

Figure 5A original blots

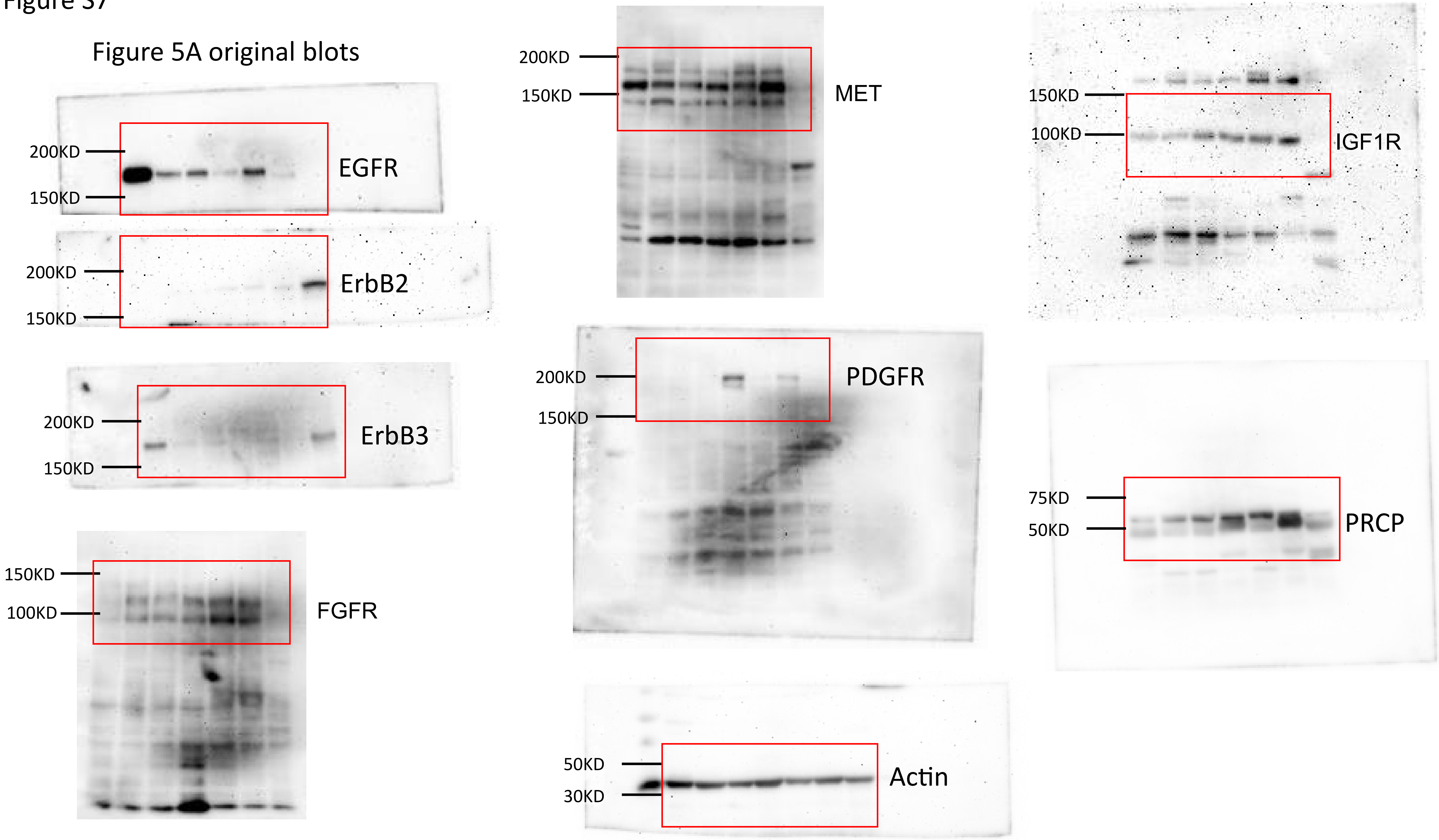

Figure S8

Figure 5B original blots

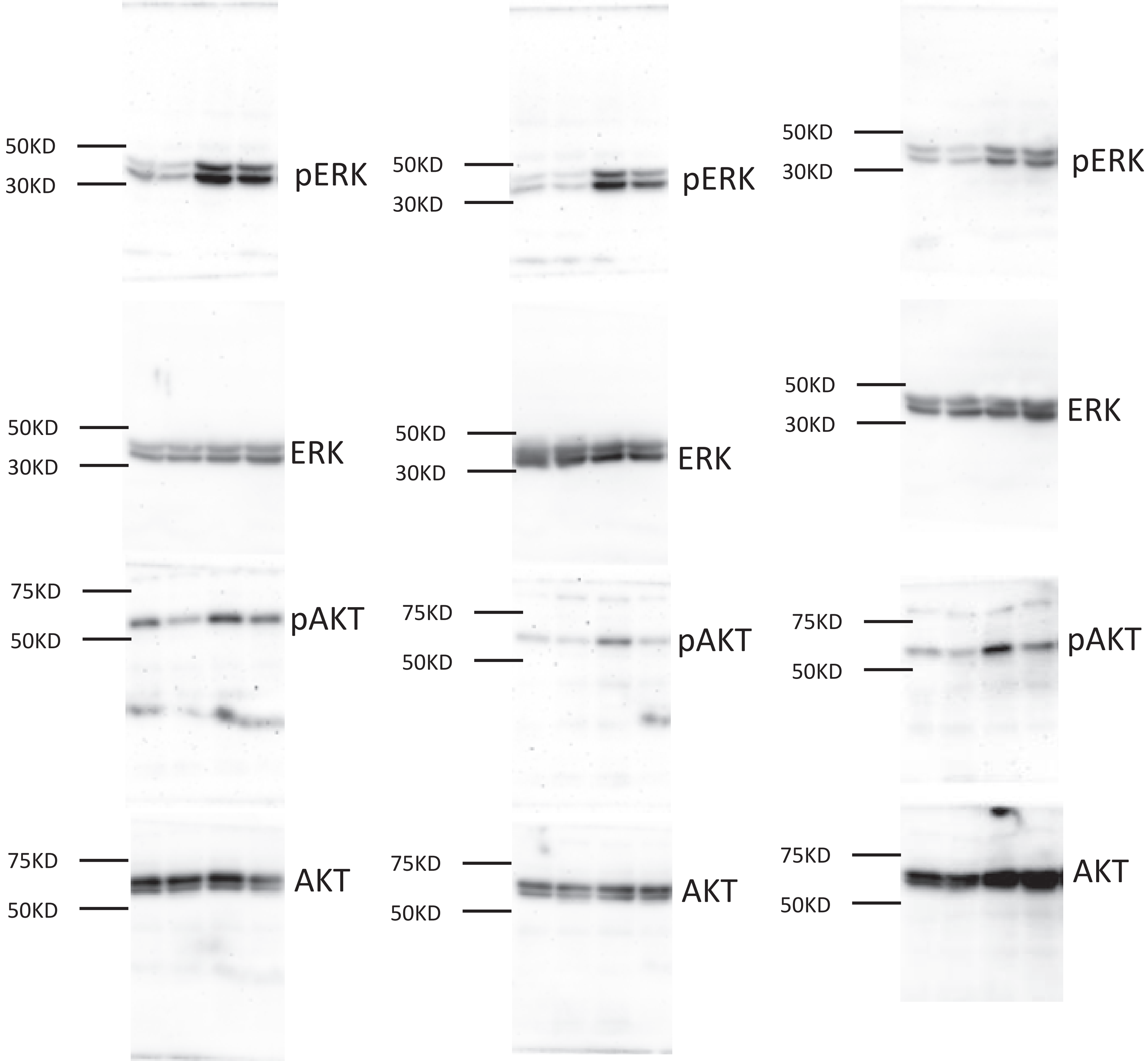

Figure S9

Figure 5C original blots

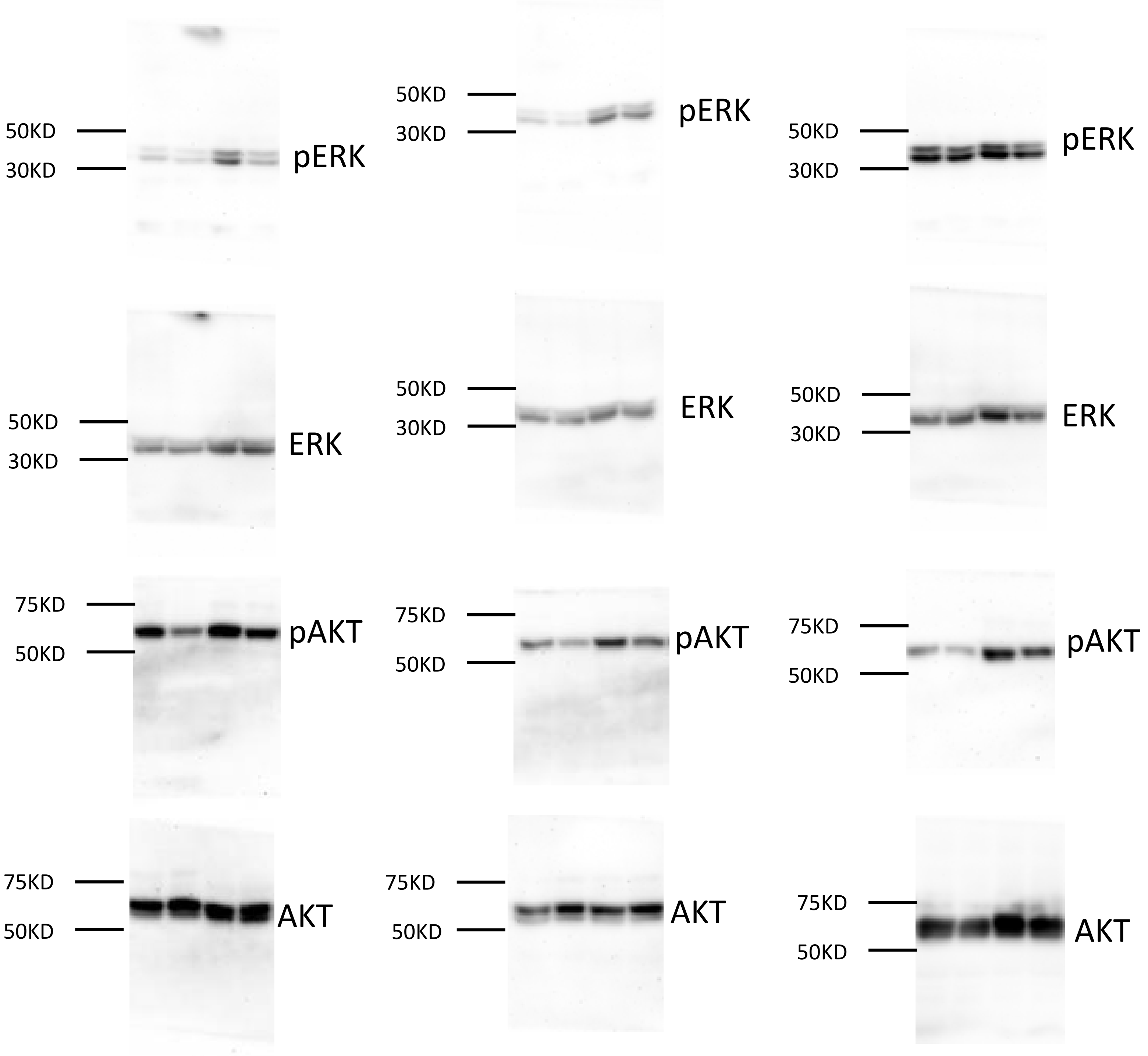

Figure S10

Figure 6A original blots

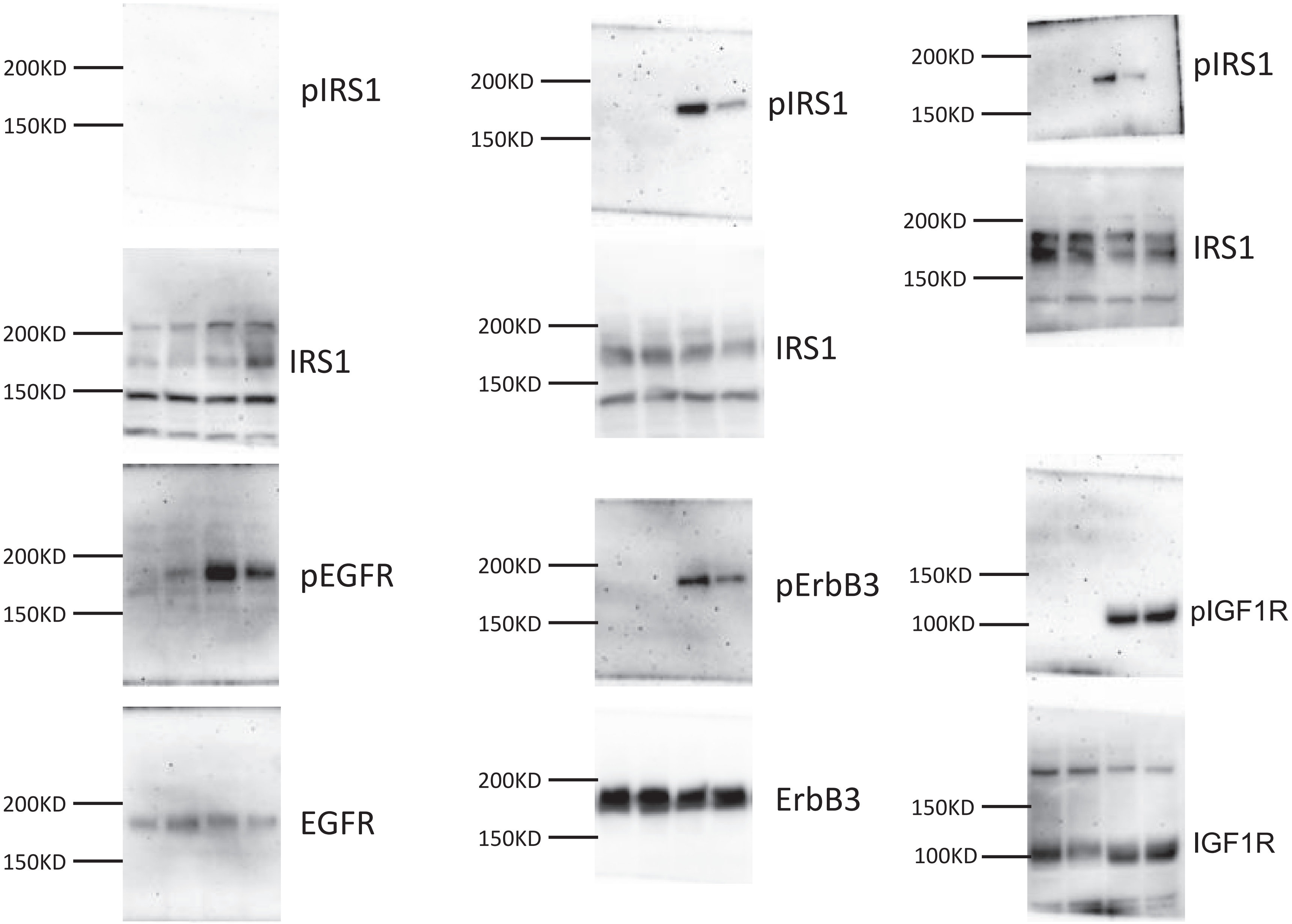

Figure S11

Figure 6B original blots

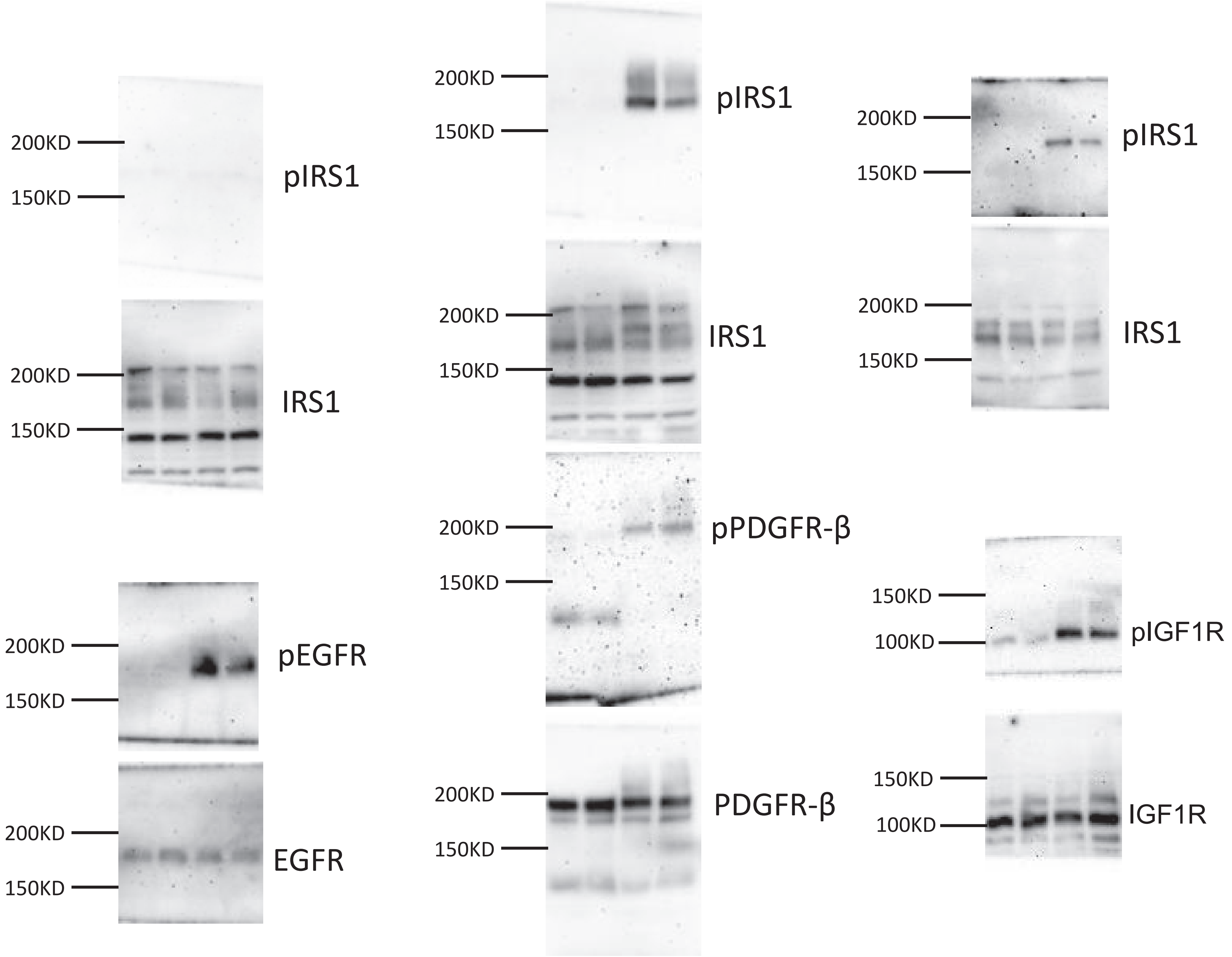

Figure S12

Figure 7A original blots

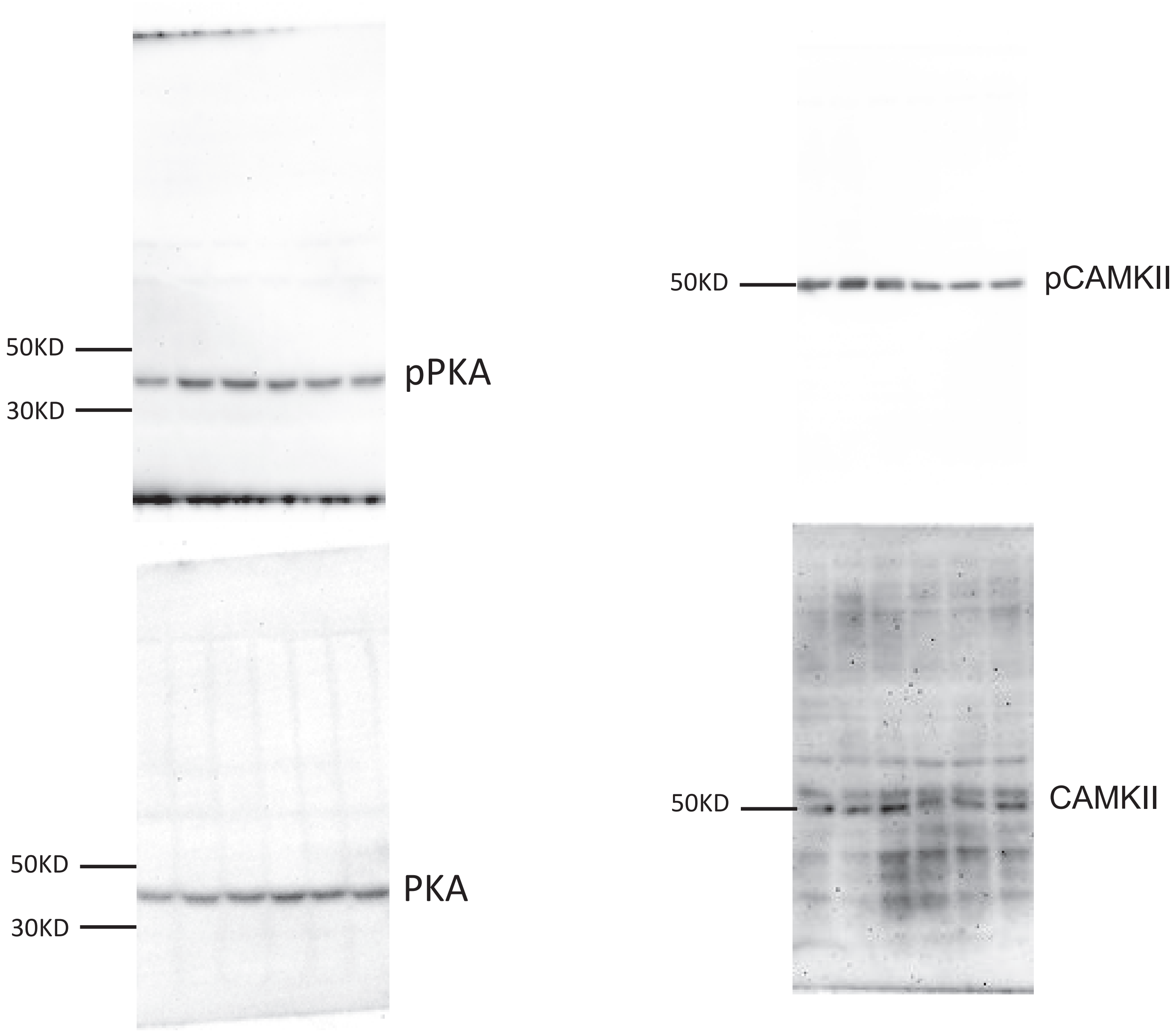

Figure S13

Figure 7B original blots

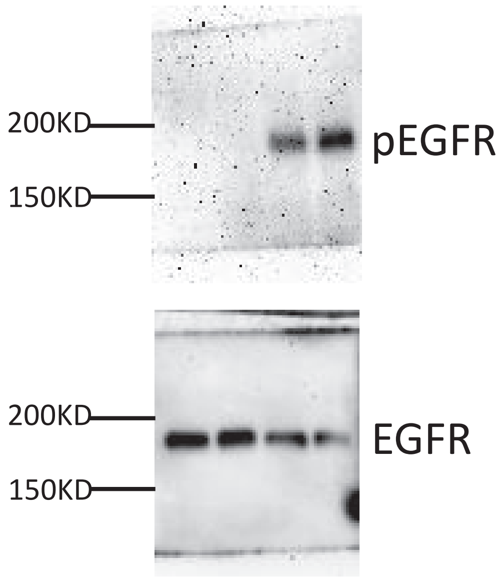

Figure 7C original blots

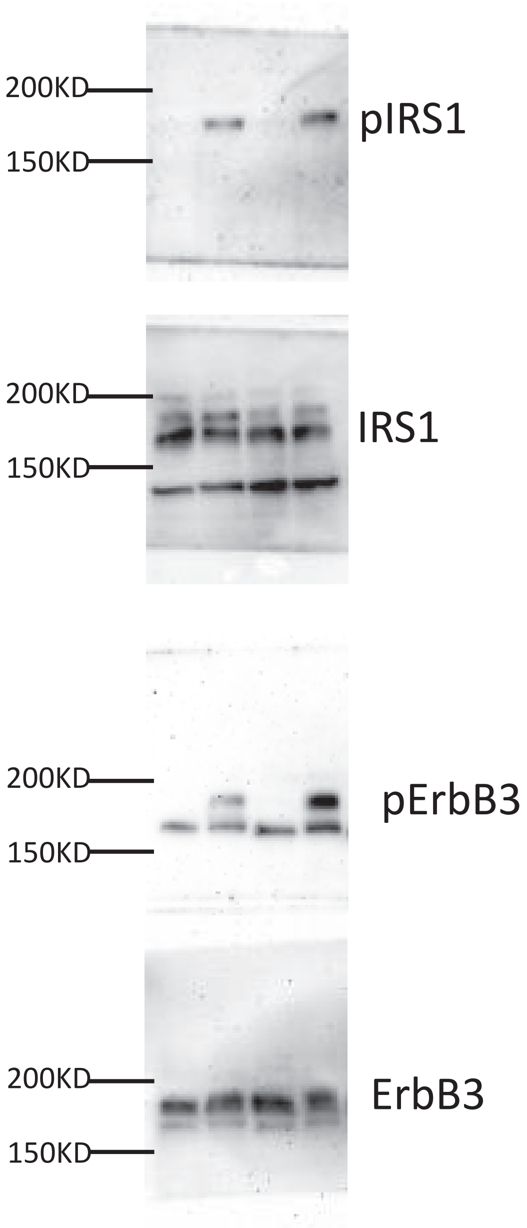

Figure 7D original blots

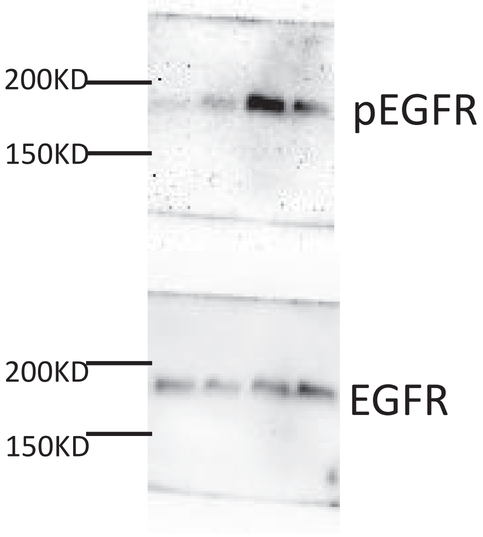

Figure 7E original blots

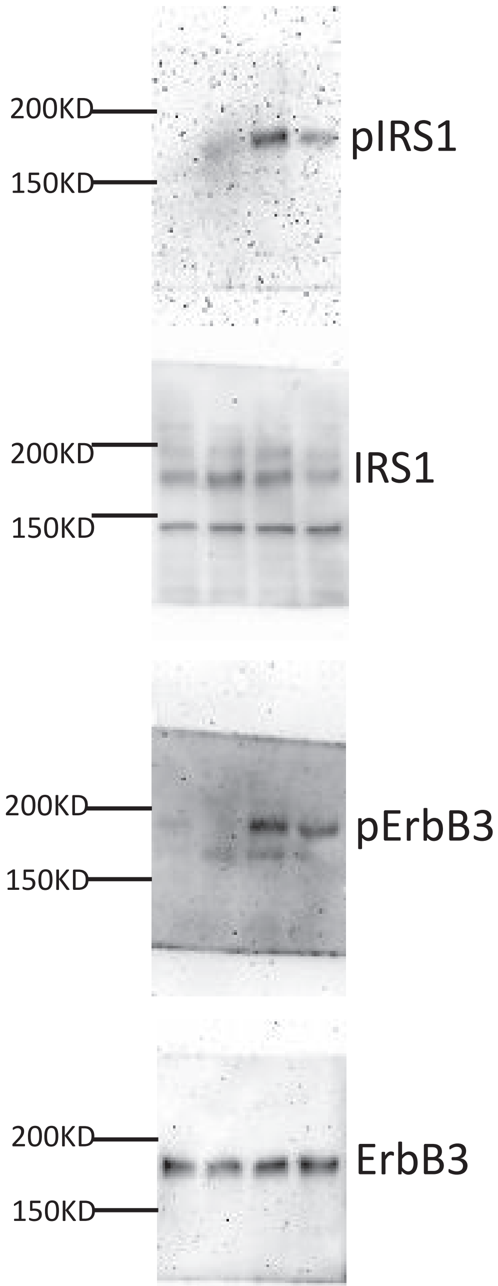

Figure S14

Figure 7F original blots

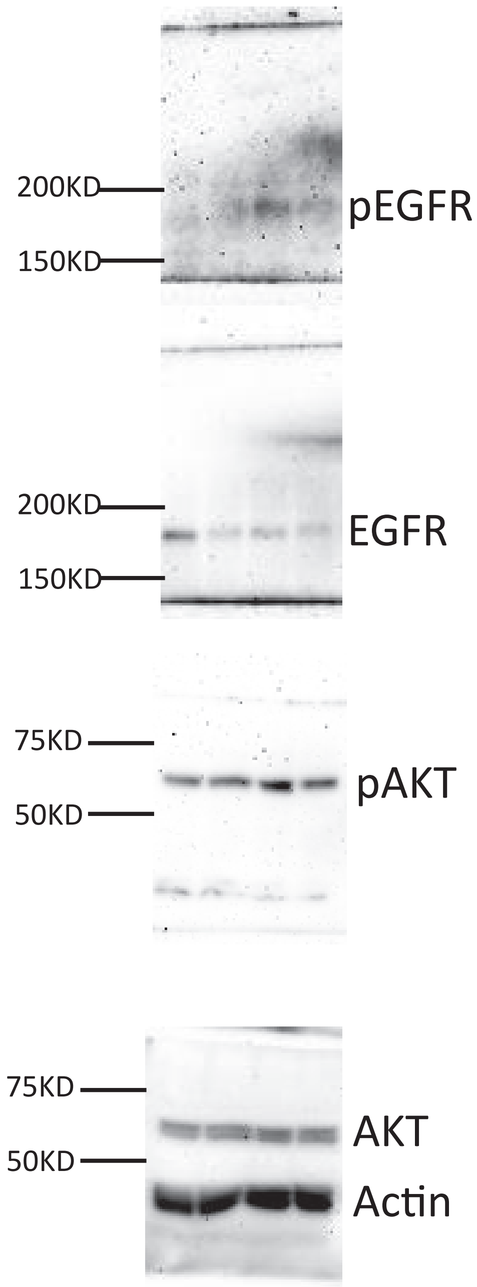

Figure 7G original blots

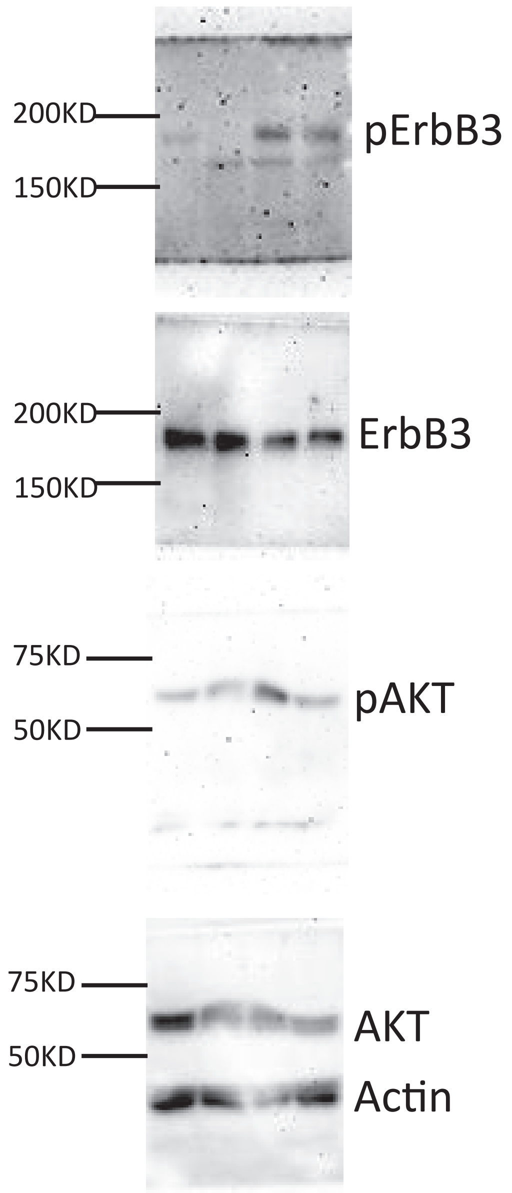

Supplement: Supplementary file 1 [file cancers-14-00739-s001.zip › cancers-1537797 Original Blots.pdf]
