# Supplementary material for: Prolyl Carboxypeptidase Maintains Receptor Tyrosine Kinase Signaling and Is a Potential Therapeutic Target in Triple Negative Breast Cancer
Source: Cancers (Basel). 2022 Jan 31;14(3):739. doi: 10.3390/cancers14030739 (PMC8833515; doi:10.3390/cancers14030739)

Supplemental figure legend

Figure S1. PRCPi induces apoptosis in TNBC cell lines. The indicated cell lines were treated with vehicle or PRCPi (5µM or 10µM) for 72 hrs. The cells were analyzed with FACS for cell cycle. Average % sub-G1 cells were presented with SD indicated.

Figure S2. PRCP cleaves Ang II and increased Ang II-mediated phosphorylation of PKA and CAMKII. A. MCF7 and B6-9 cells were serum starved and treated with Ang II (100 nM) for 5 hrs. Peptides were then precipitated from media and quantitatively analyzed for processed peptide products. Note that Ang (1-7) is increased in B6-9 cells compared with MCF7 cells. **B.** MCF7 and B6-9 cells were serum starved and treated with Ang II (100 nM) for 10 min. Lysates were immunoblotted for the indicated proteins.

Figure S1

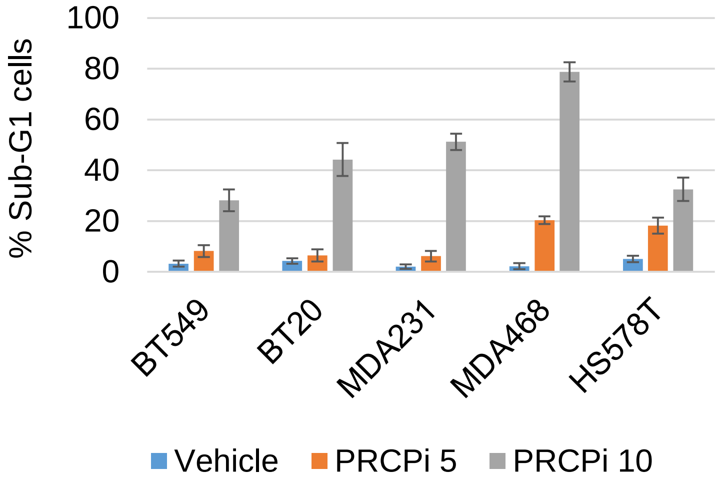

100
80
60
40
20
0
% Sub-G1 cells
BT549
BT20
MDA231
MDA468
HS578T
Vehicle
PRCPi 5
PRCPi 10

Figure S2

A.

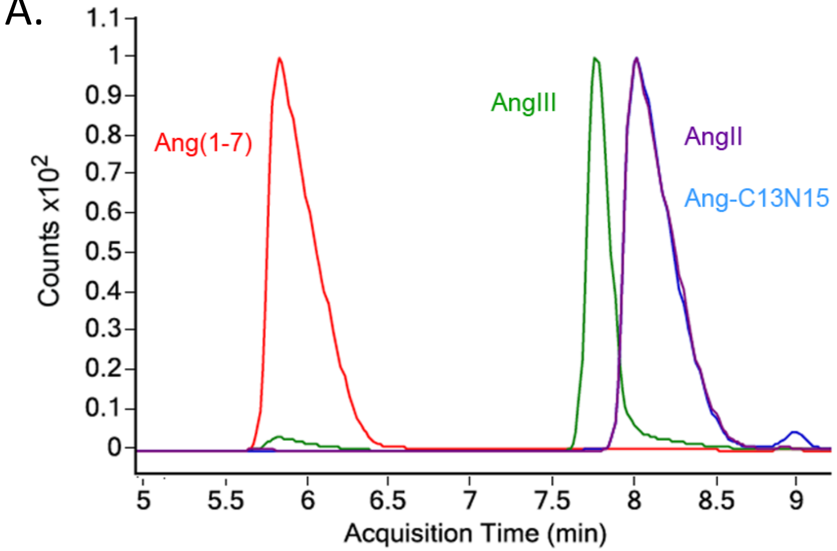

Peptide	Sequence	MCF7 0h	MCF7 5h	B6-9 0h	B6-9 5h
AngII	DRVYIHPF	100	5.7 +/- 0.5	100	8.7 +/- 0.5
AngIII	RVYIHPF		87 +/- 0.2		78.6 +/- 0.1
AngIV	VYIHPF		nd		nd
Ang(1-7)	DRVYIHP		7.4 +/- 0.3		12.7 +/- 0.4

} % of each cleavage product

B.

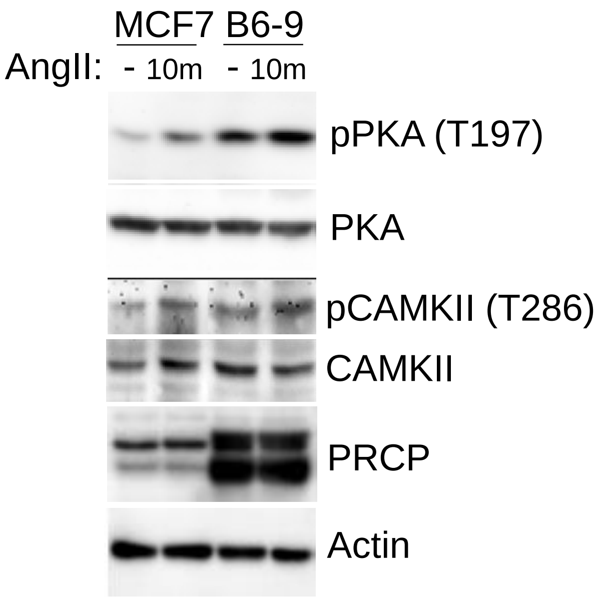

Supplement: Supplementary file 1 [file cancers-14-00739-s001.zip › cancers-1537797 supplementary.pdf]
